# Potential association of LOXL1 with peritoneal dissemination in gastric cancer possibly via promotion of EMT

**DOI:** 10.1371/journal.pone.0241140

**Published:** 2020-10-23

**Authors:** Qingjiang Hu, Takaaki Masuda, Shotaro Kuramitsu, Taro Tobo, Kuniaki Sato, Shinya Kidogami, Sho Nambara, Masami Ueda, Yusuke Tsuruda, Yosuke Kuroda, Shuhei Ito, Eiji Oki, Masaki Mori, Koshi Mimori

**Affiliations:** 1 Department of Surgery, Kyushu University Beppu Hospital, Beppu, Japan; 2 Department of Surgery and Science, Kyushu University Hospital, Fukuoka, Japan; 3 Department of Clinical Laboratory Medicine, Kyushu University Beppu Hospital, Beppu, Japan; University of Pécs Medical School, HUNGARY

## Abstract

**Background:**

Peritoneal dissemination (PD) frequently occurs in gastric cancer (GC) and is incurable. In this study, we aimed to identify novel PD-associated genes and clarify their clinical and biological significance in GC.

**Materials and methods:**

We identified *LOXL1* as a PD-associated candidate gene by *in silico* analysis of GC datasets (highly disseminated peritoneal GC cell line and two freely available GC datasets, GSE15459 and TCGA). Next, we evaluated the clinical significance of LOXL1 expression using RT-qPCR and immunohistochemistry staining (IHC) in a validation cohort (Kyushu cohort). Moreover, we performed gene expression analysis, including gene set enrichment analysis (GSEA) with GSE15459 and TCGA datasets. Finally, we performed a series of *in vitro* experiments using GC cells.

**Results:**

*In silico* analysis showed that *LOXL1* was overexpressed in tumor tissues of GC patients with PD and in highly disseminated peritoneal GC cells, relative to that in the control GC patients and cells, respectively. High expression of *LOXL1* was a poor prognostic factor in the TCGA dataset. Next, IHC showed that LOXL1 was highly expressed in GC cells. High *LOXL1* mRNA expression was associated with poorly differentiated histological type, lymph node metastasis, and was an independent poor prognostic factor in the Kyushu validation cohort. Moreover, *LOXL1* expression was positively correlated with the EMT (epithelial-mesenchymal transition) gene set in GSEA. Finally, LOXL1-overexpressing GC cells changed their morphology to a spindle-like form. LOXL1 overexpression reduced CDH1 expression; increased the expression of VIM, CDH2, SNAI2, and PLS3; and promoted the migration capacity of GC cells.

**Conclusions:**

LOXL1 is associated with PD in GC, possibly through the induction of EMT.

## Introduction

Gastric cancer (GC) is one of the most lethal malignant tumors [1] in the world. Almost 50% of recurrence is peritoneal dissemination (PD) in GC, and GC patients with PD have a poor prognosis [2]. However, the formation and the molecular characteristics of PD are still not fully understood. Thus, it is important to clarify the molecular mechanisms underlying PD in GC.

Many metastasis-related factors, such as invasion, migration, and anoikis resistance, are involved in the development of PD [3–7]. Epithelial-mesenchymal transition (EMT) is a key process for metastasis and dissemination [8] and contributes to not only migration and invasion but also anoikis resistance in cancer cells [9, 10]. Numerous intracellular signaling pathways, including TGF-β, Wnt, Notch, and hypoxia, trigger the EMT process. This process is mediated by known EMT-activating transcription factors, mainly SNAIL, SNAI2, TWIST, and ZEB1/2 [8]. A broader understanding of the regulation of EMT-activating transcription factors in cancer progression could provide valuable insights into new therapeutic targets for PD.

A series of evidence show that lysyl oxidase (LOX) is actively involved in the process of EMT [11], partly through the regulation of SNAI2 [12]. LOX expression is associated with metastasis and dissemination in GC patients [13]. The LOX family consists of five homologous members: LOX and LOX-like 1–4 (LOXL1–4). These are copper-dependent amine oxidases that catalyze covalent cross-linking of collagen and elastin fibers. *Lox*-null mice are perinatal lethal with cardiovascular dysfunction and connective tissue disorders [14]. LOXL1, LOXL2, and LOXL3 knockout mice are viable but display tissue homeostasis disorders [15–17]. LOX and LOXL2 have been well known to promote human cancer progression [18]. In contrast, relatively few data are available on the role of LOXL1 in tumorigenesis. Nevertheless, due to the structural similarity with LOX, LOXL1 may be involved in EMT and regulation of SNAI2.

Recently, we selected candidates of PD-associated genes using *in silico* analysis with GC datasets and showed that *ARL4C*, one of the candidates, could promote PD, possibly through invasive capacity [19]. Of note, *LOXL1* is also one of the previously identified candidate genes [19]. These findings provided a rationale to investigate the role of LOXL1 on EMT induction and PD in GC. In this study, we demonstrate that LOXL1 expression is associated with PD in GC, potentially via promoting EMT.

## Materials and methods

### TCGA dataset

We obtained mRNA expression data and clinical assessments of 238 GC patients from the Broad Institute’s Firehose (http://gdac.broadinstitute.org/runs/stddata__2015_06_01/data/STAD/20150601/)). The mRNA expression data (RPKM, raw count) from 238 tumor and 33 normal tissues were normalized with quantile normalization. Clinical data were available from 223 patients. Diagnosis of all GC patients was staged according to the 7^th^ edition of UICC TNM classification.

### GSE15459 dataset

GSE15459 dataset was downloaded from the Gene Expression Omnibus database (accession number GSE15459). It represents mRNA expression profiles from 200 tumor tissues (normalized with quantile normalization) and concomitant clinical data from 192 patients in Singapore (https://www.ncbi.nlm.nih.gov/geo/)). Furthermore, it contains data of 32 patients with PD (peritoneal nodules or cytology positive) and 142 patients without PD. Within this GC patient cohort, 31 patients were diagnosed with stage I, 29 patients with stage II, 72 patients with stage III, and 60 patients with stage IV, according to the 7^th^ edition of UICC TNM classification.

### Patients and sample collection from Kyushu validation cohort

This study was approved by the Ethics and Indications Committee of Kyushu University. Tumor tissues were obtained from 170 GC patients with written informed consent. These patients underwent gastrectomy at Kyushu University Beppu Hospital and affiliated hospitals from 1995 to 2009. Twenty-six patients were excluded from the initial group of 170 because they were lost to follow-up or due to the poor quality of samples. Thus, 144 GC patients were successfully enrolled in this study. All GC patients were staged according to the 7^th^ edition of UICC TNM classification. The median follow-up period for these patients was 586 days. All data for the samples, including gender, tumor size and depth of invasion, lymph node metastasis, liver metastasis, peritoneal dissemination, distant metastases (including PD and liver metastasis), clinical stage and histological grade were obtained from the clinical records. There were 48 patients with stage I, 29 patients with stage II, 33 patients with stage III, and 34 patients with stage IV. Liver metastasis (13 cases), peritoneal dissemination (19 cases), and distant metastasis (34 cases) were present at the time of the operation. We treated patients according to Japanese Gastric Cancer Treatment Guidelines. One hundred ten patients with stage I, II, and III received curative gastrectomy. The others with stage IV received palliative gastrectomy.

The tumor tissues were placed in RNAlater (Takara, Tokyo, Japan), frozen in liquid nitrogen, and stored at –80°C. Microdissections of tumor tissues were not performed because the majority of the cells in tumor tissues were of malignant origin.

### Gene Set Enrichment Analysis (GSEA)

The associations between *LOXL1* expression and previously defined gene sets were analyzed by GSEA, using GC expression profiles from GSE15459 and TCGA datasets. The biologically defined gene set was obtained at Molecular Signatures Database v5.2 (http://software.broadinstitute.org/gsea/msigdb/index.jsp). The standard name and systematic name of the gene set is HALLMARK_EPITHELIAL_MESENCHYMAL_TRANSITION and M5930, respectively. The gene set consists of 200 genes defining epithelial-mesenchymal transition in wound healing, fibrosis, and metastasis.

### Cell lines and cell culture

Human GC cell line (AGS) was purchased from American Type Culture Collection (ATCC, USA). HEK293T and MKN7 cell lines were purchased from the Japanese Collection of Research Bioresources Cell Bank (JCRB, Japan). These cell lines have been tested and authenticated using the STR-PCR method. The highly disseminated peritoneal human GC cell line (As44) and its control cell line (HSC44) were provided by Yanagihara [20, 21]. The HSC44 cell line was derived from patients with gastric scirrhous cancer. The cell line As44 showing high metastatic potential was derived from HSC44 following 12 cycles of direct orthotropic transplantation to the gastric walls of nude mice and gathering cells from the ascites. These cell lines were cultured in RPMI 1640 (Gibco, CA, USA) supplemented with 10% fetal bovine serum (FBS) at 37°C in a humidified atmosphere containing 5% CO_2_.

### RNA extraction

Total RNA was extracted from frozen tissue samples (Kyushu cohort) and cell lines using ISOGEN (NIPPON GENE, Tokyo, Japan) as previously described [22].

### Reverse transcription-quantitative PCR (RT-qPCR)

Reverse transcription was performed using M-MLV Reverse Transcriptase (Invitrogen, Carlsbad, CA, USA) as previously described [22]. Quantitative PCR was performed using LightCycler 480 SYBR Green I Master Mix (Roche, Basel, Switzerland) as previously described [22]. The following primers were used: *LOXL1*: 5’-AGGTGACCAAGTTCGCCGAG-3’ (sense) and 5’-GACGTGATAGGTGGTGGCCG-3’ (antisense); *GAPDH*: 5’-AGCCACATCGCTCAGACAC-3’ (sense) and 5’-GCCCAATACGACCAAATCC-3’ (antisense); *CDH1*: 5’-CGAGAGCTACACGTTCACGG-3’ (sense) and 5’-AGTCCCAGGCGTAGACCAAG-3’ (antisense); *VIM*: 5’-TACAGGAAGCTGCTGGAAGG-3’ (sense) and 5’-ACCAGAGGGAGTGAATCCAG-3’ (antisense). *SNAI2*: 5’- CAACGCCTCCAAAAAGCCAA-3’ (sense) and 5’- ACTCACTCGCCCCAAAGATG-3’ (antisense). *ZEB1*: 5’-TTTTTCCTGAGGCACCTGAA-3’ (sense) and 5’-AAAATGCATCTGGTGTTCCAT-3’ (antisense). *PLS3*: 5’-CCTTCCGTAACTGGATGAACTC -3’ (sense) and 5’-GGATGCTTCCCTAATTCAACAG-3’ (antisense). We calculated the mRNA expression levels using the standard curve method, as previously reported [23]. After the PCR amplification, the cycle number of each sample was used as a crossing point value. A standard curve was produced by measuring the crossing point of each standard value (5-fold serially diluted cDNAs from Human Universal Reference Total RNA (Clontech Laboratories, Palo Alto, CA, USA)) and plotting them against the logarithmic value of each concentration. Then the mRNA expression level of each sample was calculated by setting its crossing points to the standard curve. The mRNA expression levels of *LOXL1*, *CDH1*, *VIM*, *SNAI2*, *ZEB1*, and *PLS3* were also normalized to that of *GAPDH* mRNA. GC cell lines (LOXL1-overexpressing AGS, control AGS, As44, and HSC44) and GC tissues from the Kyushu validation cohort were used for RT-qPCR. All experiments were replicated at least three times in this study.

### DNA microarray of highly disseminated peritoneal GC cell line

We used mRNA expression data (DNA microarray) from highly disseminated peritoneal human GC cell line (As44) and its control cell line (HSC44), which we previously reported [22].

### Establishment of LOXL1-overexpressing stable cell line

A full-length cDNA insert of human LOXL1 was amplified by PCR and subcloned downstream of the CMV promoter, resulting in the generation of the pCSII-CMV-LOXL1 vector (at 5′ NheI and 3′ XbaI sites). Lentiviruses were generated by transfection of HEK293T cells with pCMV-VSV-G-RSV-Rev, pCAG-HIVgp, and either pCSII-CMV-LOXL1 or pCSII-CMV-MCS (empty) plasmid DNAs using Lipofectamine 2000 (Invitrogen, MA, USA) following the manufacturer's protocol. Forty-eight hours after transfection, the lentivirus-containing supernatant was collected and passed through a 0.45 μm filter. Infections were subsequently carried out by incubating the AGS cells in medium containing the lentiviral supernatant for 48 hours. The final LOXL1-overexpressing stable and control (mock) cell lines were established by selection using Zeocin (Thermo Fisher Scientific, MA, USA).

### Western blot analysis

Total protein lysates (35 μg), obtained from GC cells, were electrophoresed on 10% polyacrylamide gels and then electroblotted onto Immobilon-P Transfer Membranes (Merck Millipore, MA, USA) at 70 V for four hours at 4°C. The following specific antibodies (all at a dilution of 1:1000) were used: primary rabbit polyclonal antibodies against LOXL1 (H00004016-D01P, Abnova); anti-SNAI2 antibody (ab27568, Abcam, Cambridge, UK); anti-ZEB1 antibody (ab124512, Abcam, Cambridge, UK); anti-PLS3 antibody (sc-166555, Santa Cruz Biotechnology, TX, USA); primary mouse monoclonal antibody against β-actin (sc-47778, Santa Cruz Biotechnology). Expression of LOXL1, SNAI2, and PLS3 proteins was normalized to the expression of β-actin protein. An ImageQuant LAS 4000 Mini system (GE Healthcare Japan) was used to detect antigen-antibody reactions.

### Immunofluorescence analysis

A primary mouse monoclonal antibody against CDH2 (ab98952, Abcam, dilution 1:200) and Alexa Fluor 594-conjugated anti-mouse immunoglobulin were used to stain CDH2 in LOXL1-overexpressing and control cells. SlowFade® Gold Antifade Mountant with DAPI was used for mounting microscope slides and identifying nuclei (Invitrogen, MA, USA). Fluorescent images were observed under a fluorescence microscope (BZ-X700, Keyence, Osaka, Japan).

### Immunochemistry staining

Immunohistochemical analysis of LOXL1 was performed on formalin-fixed, paraffin-embedded specimens from five GC patients from Kyushu University Hospital using the avidin-biotin-peroxidase method (LSAB2 kit; Dako, Kyoto, Japan). The LOXL1 primary antibody (H00004016-D01P, Abnova) was used at a dilution of 1:100. Histological analysis was independently performed by an experienced research pathologist at Kyushu University.

### Wound-healing assay

LOXL1-overexpressing and control cells in six-well plates were scratched three times with a sterile 10 μl pipette tip to form parallel lines and subsequently washed with PBS for removal of non-adherent cells. The six-well plates were then incubated at 37°C, 5% CO2 for 24 and 48 h, and the same wound areas were observed and photographed under an inverted microscope (BZ-X700, Keyence, Osaka, Japan). The distance of the scratch closure was examined at 0, 24, and 48 h. GC cell lines (LOXL1-overexpressing AGS and control AGS) were used for wound-healing assays, which were replicated six times in this study.

### Statistical analysis

For clinical analyses, cases were divided into two groups using the minimum *p*-value approach based on *LOXL1* expression level. The minimum *p*-value approach is a comprehensive method to identify the optimal risk separation cut-off point in continuous gene expression measurements for survival analysis in multiple datasets [24]. Associations between the variables were tested with Student's t-test, Chi-squared test (likelihood-ratio test), and Fisher’s exact test. The degree of linearity was estimated by Pearson’s correlation coefficient. Overall survival was estimated using the Kaplan-Meier method, and survival curves were compared using the log-rank test. Univariate and multivariate analyses were performed using the Cox regression model to identify independent variables predictive of overall survival. A two-sided value of P < 0.05 was considered significant. Data analyses were performed using JMP Pro 14 software (SAS Institute, Cary, NC, USA) and R software version 3.1.1 (The R Foundation).

## Results

### *LOXL1* is a PD-associated candidate gene in GC

**Fig 1:**
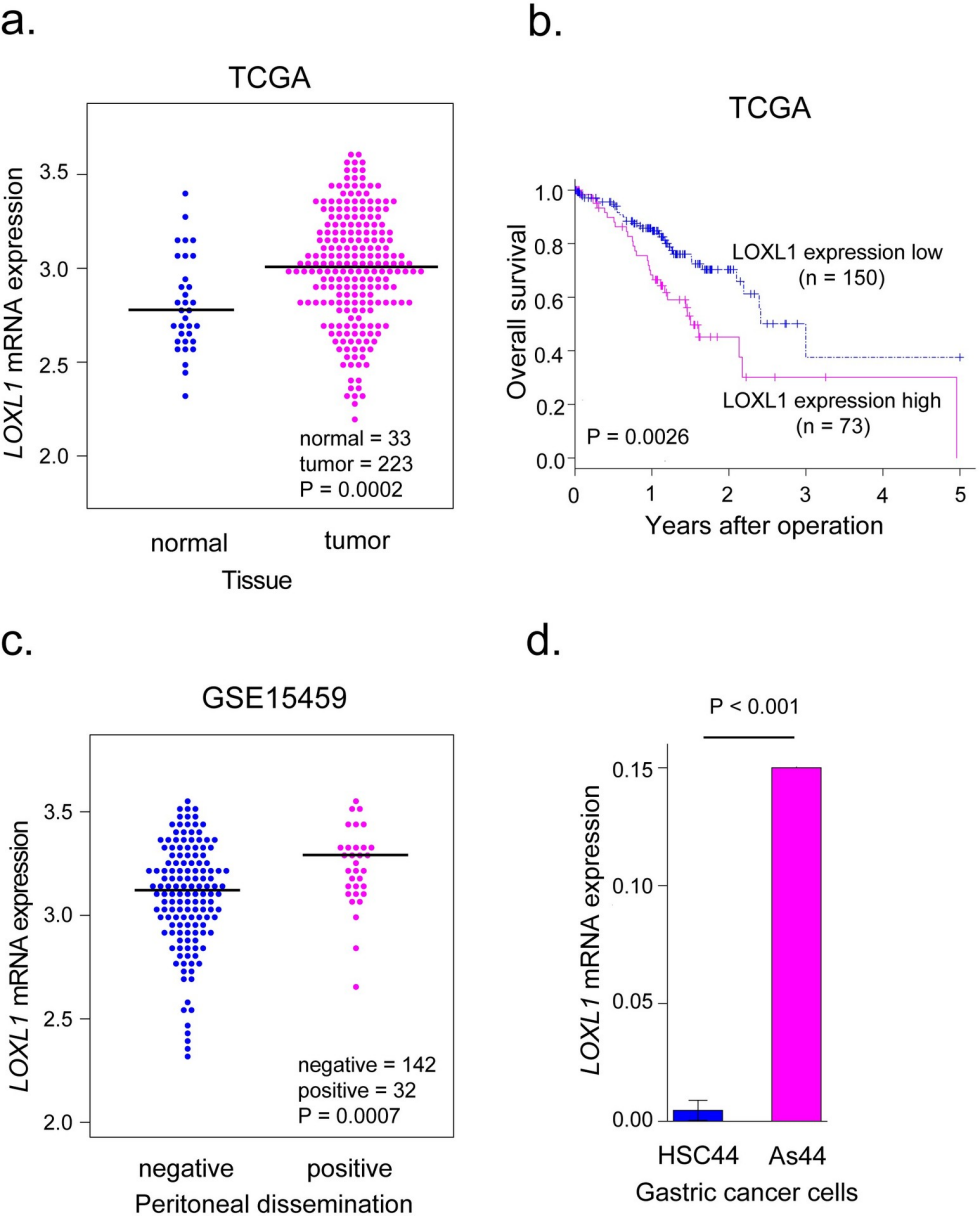
*LOXL1* was identified as a PD-associated candidate gene in GC. **a.** *LOXL1* mRNA expression in 223 tumor and 33 normal tissues of GC patients from the TCGA dataset. **b.** Kaplan-Meier survival curve of 223 GC patients from the TCGA dataset based on *LOXL1* mRNA expression; log-rank test, n = 223, P < 0.01. **c.** *LOXL1* mRNA expression in GC patients with PD compared with that in GC patients without PD from the GSE15459 dataset. PD negative, n = 142, PD positive, n = 32; Student's t-test P < 0.001. **d.** *LOXL1* mRNA expression in highly disseminated peritoneal GC cells (As44) compared with the control cells (HSC44), examined by RT-qPCR; Student's t-test P < 0.001.

In our previous study, we identified 25 PD-associated candidate genes by *in silico* analysis of GC datasets (GSE15459, highly disseminated peritoneal GC cell line, and TCGA) [19]. The PD-associated candidate genes satisfied four criteria as follows. The genes had to be overexpressed in highly-disseminated peritoneal GC cell lines. Secondly, the genes had to be overexpressed in GC patients with PD compared to GC patients without PD. Finally, the genes had to be overexpressed in tumor tissues of GC patients compared to normal tissues, and high expression of the gene had to be a poor prognostic factor in GC patients. *LOXL1* was identified as one of the PD-associated candidate genes [19]. *LOXL1* expression was higher in tumor tissues than in normal gastric tissues (Fig 1A), and high expression of *LOXL1* was a poor prognostic factor in the TCGA dataset (Fig 1B). Along with that, the expression level of *LOXL1* was higher in the tumor tissues of GC patients with PD than in GC patients without PD in the GSE15459 dataset (Fig 1C). Moreover, *LOXL1* was overexpressed in highly disseminated peritoneal GC cells compared to the control GC cells (Fig 1D).

### Clinicopathological analysis of *LOXL1* expression in Kyushu validation cohort

**Fig 2:**
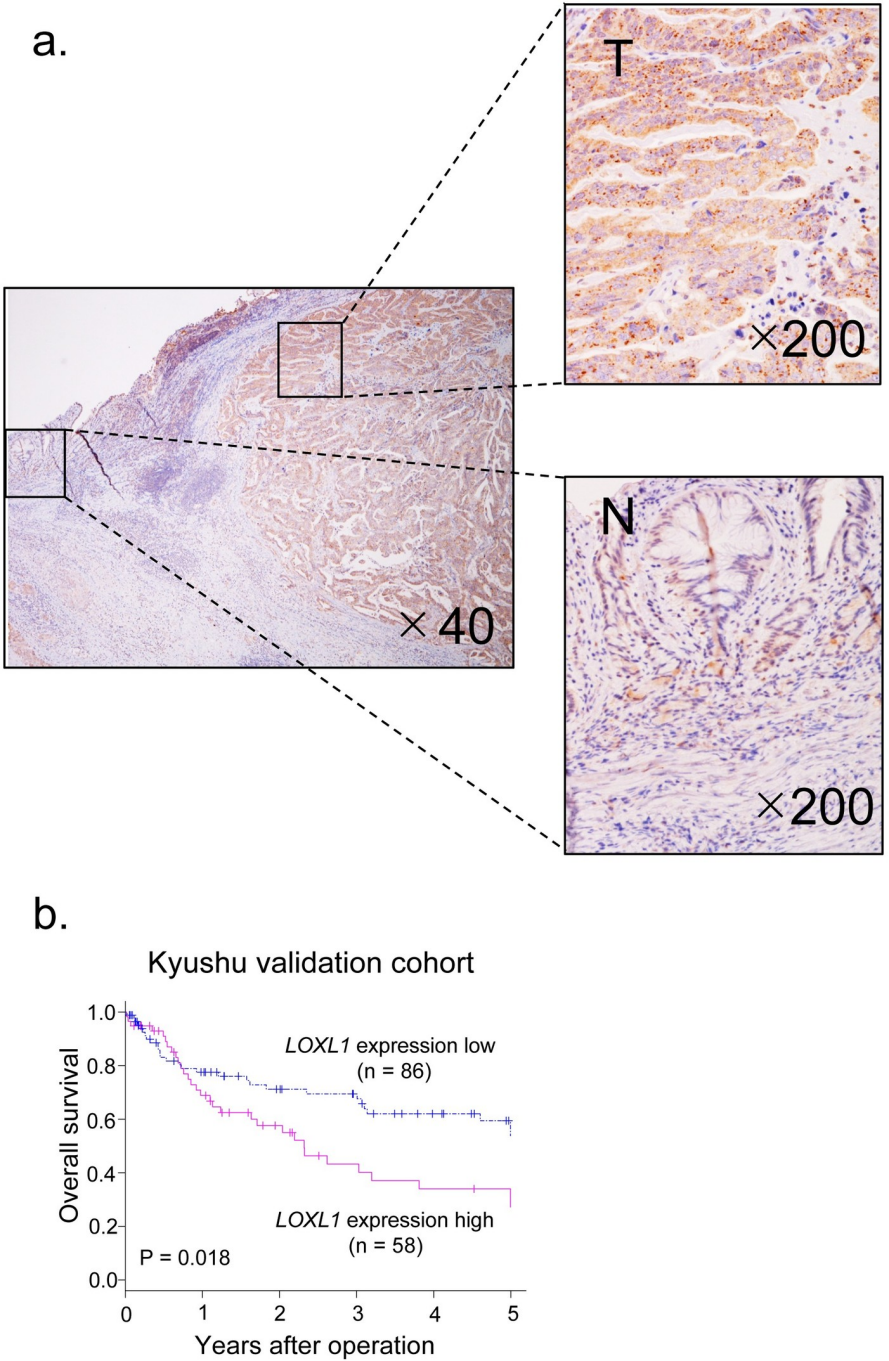
*LOXL1* expression was found to be a prognostic factor for overall survival of GC patients. **a.** Immunochemistry staining of LOXL1 in representative GC tissues from the Kyushu validation cohort. N: normal tissue, T: Tumor tissue; original magnification, ×40, ×200. **b.** Kaplan-Meier survival curve of 144 GC patients from the Kyushu validation cohort based on *LOXL1* mRNA expression; log-rank test, n = 144, P < 0.05.

We analyzed LOXL1 expression by IHC (n = 5) and RT-qPCR (n = 144) in GC tissues of the Kyushu validation cohort. IHC showed that LOXL1 was highly expressed in tumor cells from 4 out of 5 GC tissues (Fig 2A and S1 Fig). Moreover, LOXL1 immunostaining intensity was positively associated with *LOXL1* mRNA expression level in GC patients (S1 Fig). Based on the *LOXL1* mRNA expression level, we divided GC patients into high and low *LOXL1* expression groups. The clinicopathological analysis showed that the high *LOXL1* expression group was significantly associated with a poorly differentiated histological type (Chi-squared test, P < 0.05, Table 1) and lymph node metastasis (Chi-squared test, P < 0.05, Table 1). However, there was no statistically significant difference in PD between high and low *LOXL1* expression groups in the Kyushu validation cohort, although the high *LOXL1* expression group had around twice more PD cases than the low expression group (Chi-squared test, P = 0.15, Table 1).

**Table 1:** *LOXL1* mRNA expression and clinicopathological factors of GC cases in the Kyushu validation cohort (n = 144).

Factors	High expression	Low expression	
	(n = 58)	(n = 86)	
	Number (%)	Number (%)	P-value
Age (mean ± SD)	64.9 ± 11.8	67.8 ± 11.3	0.15
Gender			
Female	22 (38)	32 (37)	0.93
Histological type			
MUC, POR, SIG	34 (59)	32 (37)	0.01
Depth of tumor invasion			
≥ SE	5 (9)	11 (13)	0.43
Lymph node metastasis			
(+)	44 (76)	49 (57)	0.02
Liver metastasis			
(+)	4 (7)	9 (10)	0.46
Peritoneal dissemination			
(+)	11 (19)	9 (10)	0.15
Distant metastases			
(+)	15 (26)	19 (22)	0.60
pStage			
≥ III	29 (50)	38 (44)	0.49

*SD* standard deviation, *MUC* mucinous adenocarcinoma, *POR* poorly differentiated adenocarcinoma, *SIG* signet-ring cell carcinoma, *SE* serosa.

### Prognostic analysis of *LOXL1* expression in Kyushu validation cohort

Next, we performed a prognostic analysis of *LOXL1* expression in the Kyushu validation cohort. We divided the total cases into high and low *LOXL1* expression groups using the minimum *p*-value approach based on the *LOXL1* expression level, as described in the Materials and methods section under “statistical analysis” [24]. The overall survival of the high *LOXL1* expression group was significantly reduced than that of the low expression group (Fig 2B).

Furthermore, we performed a multivariate analysis with four variables (*LOXL1* expression, depth of tumor invasion, lymph node metastasis, distant metastases) among all eight variables affecting overall survival in univariate analyses in the Kyushu validation cohort. We found that high *LOXL1* expression was an independent prognostic factor for poor outcome (P < 0.05, HR = 1.72, 95% CI = 1.00–2.97; Table 2).

**Table 2:** Univariate and multivariate analyses of clinicopathological factors affecting overall survival in GC cases from the Kyushu validation cohort (n = 144).

	Univariate analysis		Multivariate analysis	
Variable	Hazard ratio (CI)	P-value	Hazard ratio (CI)	P-value
*LOXL1* expression	1.88	<0.05	1.72	<0.05
High	(1.12–3.18)		(1.00–2.97)	
Age	0.54	<0.05		
≥65 years	(0.32–0.90)			
Gender	0.80	0.45		
Female	(0.46–1.42)			
Histological type	1.25	0.40		
MUC, POR, SIG	(0.74–2.10)			
Depth of tumor invasion	4.08	<0.001	2.52	<0.01
≥ SE	(2.18–7.62)		(1.29–4.93)	
Lymph node metastasis	6.80	<0.001	4.53	<0.001
(+)	(2.91–15.89)		(1.90–10.84)	
Liver metastasis	6.63	<0.001		
(+)	(3.18–13.80)			
Peritoneal dissemination	4.55	<0.001		
(+)	(2.42–8.56)			
Distant metastases	7.68	<0.001	4.57	<0.001
(+)	(4.31–13.67)		(2.48–8.43)	
pStage	6.05	<0.001		
≥ II	(3.29–11.11)			

*CI* confidential interval, *MUC* mucinous adenocarcinoma, *POR* poorly differentiated adenocarcinoma, *SIG* signet-ring cell carcinoma, *SE* serosa.

### LOXL1 overexpression may induce EMT in GC cells

**Fig 3:**
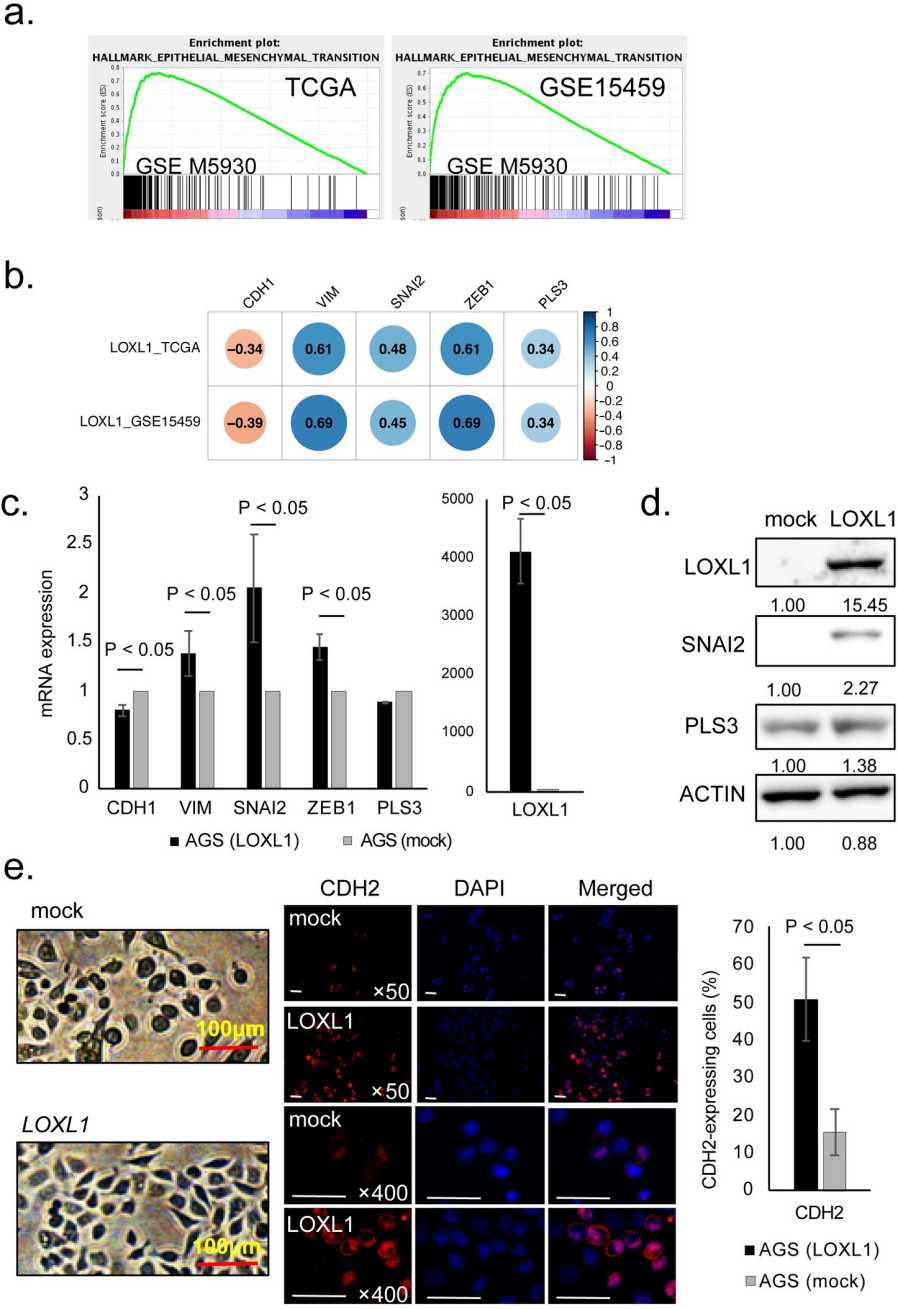
LOXL1 overexpression may induce EMT in GC cells. **a.** GSEA of GC cases from TCGA and GSE15459 datasets. TCGA dataset: NES = 2.08, FDR q-value < 0.001; GSE15459 dataset: NES = 2.01, FDR q-value < 0.05. **b.** Correlations between *LOXL1* and EMT markers (*CDH1*, *VIM*, *SNAI2*, *ZEB1*, and *PLS3*) expression levels in GC patients from TCGA and GSE15459 datasets. Positive correlation was represented in blue and negative correlation was represented in red. The darker the color, the higher the correlation was (P < 0.05). The value in the box represented the correlation coefficient. **c.** The mRNA expressions of *CDH1*, *VIM*, *SNAI2*, *ZEB1*, *PLS3*, and *LOXL1* were analyzed by RT-qPCR in LOXL1-overexpressing AGS cells and control cells. The expression levels were expressed as the values relative to the mRNA expression in the control cells. Student’s t-test P < 0.05. **d.** Western blot analysis of LOXL1, SNAI2, PLS3, and ACTIN protein expression in LOXL1-overexpressing and control cells. **e.** The shape of LOXL1-overexpressing and control cells (left panel); and immunofluorescence analysis of CDH2 in LOXL1-overexpressing and control cells (right panel). Original magnification, ×50, ×400. Student’s t-test P < 0.05. Scale bar is 100μm.

To investigate the effect of LOXL1 expression on PD, we performed GSEA using TCGA and GSE15459 datasets. The GSEA revealed that high expression of *LOXL1* positively correlated with the EMT gene set (Fig 3A). Moreover, we evaluated the correlations between *LOXL1* mRNA expression and the well-known EMT markers (*CDH1*, *VIM*, *SNAI2*, *ZEB1*, and *PLS3*) [25–27]. *LOXL1* mRNA expression correlated negatively with *CDH1*, and positively with the mRNA expression levels of *VIM*, *SNAI2*, *ZEB1*, and *PLS3* both in TCGA and GSE15459 datasets (Fig 3B). We also examined the mRNA expression of *LOXL1* and above EMT markers (*CDH1*, *VIM*, *SNAI2*, *ZEB1*, and *PLS3*) using DNA microarray data of HSC44 and As44 cells. The mRNA expression of *LOXL1* and *VIM* were remarkably higher in As44 cells than that in HSC44 cells (S2 Fig).

Next, we established a LOXL1-overexpressing cell line using AGS cells. We examined the mRNA expression of *LOXL1* and above EMT markers in LOXL1-overexpressing AGS cells and the control cells by RT-qPCR. The mRNA expression of *CDH1* in LOXL1-overexpressing AGS cells was lower than in the control cells (Fig 3C). Additionally, mRNA expression of *VIM*, *SNAI2*, *ZEB1* in LOXL1-overexpressing AGS cells was higher than in the control cells. Furthermore, we examined the protein levels of SNAI2, ZEB1, and PLS3 in LOXL1-overexpressing AGS cells and the control cells. SNAI2 and PLS3 were upregulated in LOXL1-overexpressing AGS cells compared with the control cells (Fig 3D). However, western blot showed that ZEB1 was not expressed in both LOXL1-overexpressing AGS cells and the control cells (S2 Fig). Finally, we found that the morphology of LOXL1-overexpressing AGS cells changed to a spindle-like form (Fig 3E, left panel). The percentage of CDH2-expressing cells was significantly higher in LOXL1-overexpressing AGS cells than in the control cells (Fig 3E, right panel). These results indicated that LOXL1 overexpression may induce EMT in GC cells.

### LOXL1 overexpression promoted cell mobility in GC

**Fig 4:**
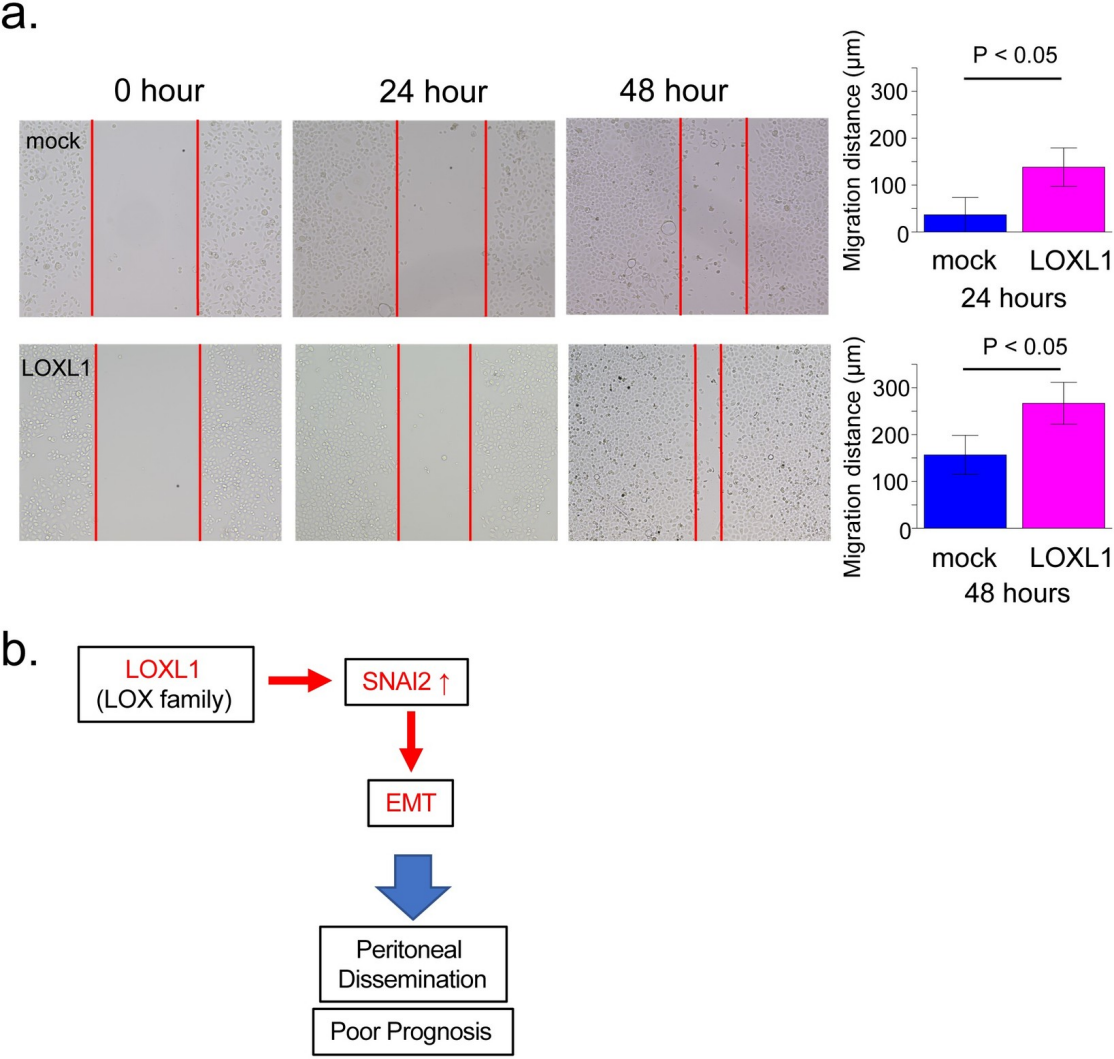
LOXL1 overexpression promoted migration capacity of GC cells. **a.** Images of *in vitro* wound-healing assay (left panel), and migration distance of LOXL1-overexpressing and control cells (right panel). Student's t-test P < 0.05. **b.** Schematic representation of the mechanism of PD promotion by LOXL.

Cell migration is required for the formation of PD in GC [3]. Moreover, LOXL1 overexpression may induce EMT that is well known to promote tumor cell migration [25]. Thus, to evaluate the effect of LOXL1 on GC cell motility, we performed a wound-healing assay. The distance of the scratch closure was shorter in LOXL1-overexpressing AGS cells than in the control cells after incubation for 24 and 48 h (Fig 4A). This result indicates that LOXL1 overexpression promoted GC cell mobility.

### *LOXL1* mRNA did not correlate with DNA copy number in GC samples

DNA amplification is a major mechanism that drives gene overexpression. To investigate whether *LOXL1* overexpression was induced by *LOXL1* DNA amplification in GC tissue samples, we calculated Pearson-Correlation between DNA copy number variation and mRNA expression of *LOXL1* using a publicly available online portal, LinkedOmics, that includes multi-omics data from all 32 TCGA cancer types [28]. However, *LOXL1* mRNA did not correlate with DNA copy number variations in GC patients (S3 Fig), suggesting that DNA amplification may not be the reason for *LOXL1* overexpression in GC.

## Discussion

In this study, we identified *LOXL1* as a PD-associated candidate gene in GC by *in silico* analysis of GC datasets. We found that high *LOXL1* expression was associated with poorly differentiated histological type, lymph node metastasis, and poor prognosis in GC from the Kyushu validation cohort. Furthermore, we demonstrated that LOXL1 overexpression may induce EMT, promoting cell mobility, and upregulating SNAI2 expression in GC cells. These experimental and clinical observations suggest that LOXL1 is associated with PD, potentially via promoting EMT in GC cells (Fig 4B).

LOXL1 is an extracellular copper-dependent amine oxidase [11]. It is known that LOXL1 is expressed in stromal cells and promotes non-small cell lung cancer tumorigenesis by extracellular matrix remodeling [29]. Interestingly, a recent study reported that LOXL1 is overexpressed in GC cells, and that high LOXL1 expression is a poor prognostic factor in GC patients [30]. Our clinical findings were consistent with this report, suggesting that LOXL1 may affect tumor progression in GC cells.

Moreover, the structural homolog of LOXL1, LOX, transcriptionally regulates SNAI2 expression by transactivating the SNAI2 promoter in human cancer cells [12]. SNAI2 is a well-known EMT marker. During EMT, epithelial cells lose their epithelial markers, upregulate mesenchymal markers, and change their cell shape [8]. We found that LOXL1 overexpression downregulated CDH1 expression and upregulated the expression of VIM, CDH2, PLS3, and SNAI2 in GC cells. Moreover, LOXL1-overexpressing AGS cells changed their morphology to a spindle-like form and increased cell mobility. These results demonstrated that LOXL1 overexpression may induce EMT in GC cells. SNAI2 belongs to the SNAIL superfamily of zinc-finger transcription factors and can induce EMT through the repression of epithelial genes by its binding to E-box DNA sequences [31, 32]. ZEB1 was also reported as one of the mediators of SNAI2-induced EMT in melanoma [33]. In this study, LOXL1 overexpression upregulated the expression of SNAI2. However, no changes were found in ZEB1 protein levels between LOXL1-overexpressing cells and control cells. Thus, ZEB1 may not be involved in LOXL1-induced EMT in GC cells. LOXL1 may induce EMT, possibly through upregulating SNAI2 expression in GC cells. The mechanism underlying LOXL1-induced EMT via upregulating SNAI2 needs further investigation.

*In silico* analysis of GC datasets showed that high *LOXL1* expression was significantly associated with PD, although there was no statistically significant difference in PD between high and low *LOXL1* expression group in the Kyushu validation cohort. This finding may be due to the small size of the Kyushu validation cohort. The clinicopathological analysis showed that high *LOXL1* expression was associated with poorly differentiated histological type and lymph node metastasis. The tumor cells with poorly differentiated histological type are considered to have high invasive potential, which promotes metastasis, including PD or lymph node metastasis. Our experimental data supported the result of these clinical analyses, suggesting that LOXL1 may play a critical role not only in PD but also in lymph node metastasis in GC patients.

The mechanism of LOXL1 upregulation in GC cells remains unknown. In our study, *LOXL1* mRNA did not correlate with *LOXL1* DNA copy number in GC patients, suggesting that *LOXL1* may be regulated by other mechanisms such as TGF-β signaling [34]. For further confirmation of LOXL1 impact in GC pathogenesis, more experiments are needed.

In conclusion, our results showed that LOXL1 was associated with PD in GC, possibly through the induction of EMT. Thus, LOXL1 may be a biomarker and a potential therapeutic target for PD in GC.

## Supporting information

S1 Fig Immunochemistry staining of LOXL1 was performed in GC tissue. Immunochemistry staining of LOXL1 in 5 GC tissues from the Kyushu validation cohort. Original magnification, ×40, ×100, ×400; LOXL1 immunostaining intensity in tumor cells from the 5 GC tissues were classified into three levels (low, medium, and high).
(ZIP)

S2 Fig The expression of EMT markers were examined in GC cells. **a.** The ratios of *LOXL1* expression, *CDH1* expression, *VIM* expression, *SNAI2* expression, *ZEB1* expression, and *PLS3* expression in As44 cells relative to HSC44 cells. **b.** Western blot analysis of LOXL1, ZEB1, and ACTIN protein expression in LOXL1-overexpressing AGS cells, the control AGS cells, and MKN7 cells.
(TIF)

S3 Fig *LOXL1* mRNA did not correlate with DNA copy number. Correlation between *LOXL1* mRNA expression and DNA copy number in GC patients.
(TIF)

S4 Fig Raw images of western blot. (ZIP)

S1 Table Clinicopathological factors of GC cases with top 10 high and top 10 low LOXL1 mRNA expression in the Kyushu validation cohort (n = 20, Chi-squared test for P-value). (DOCX)

S1 File MTT assay of LOXL1-overexpressing AGS cells and the control cells. (TIF)
