# Supplementary figures and images for: Potential association of LOXL1 with peritoneal dissemination in gastric cancer possibly via promotion of EMT
Source: PLoS One. 2020 Oct 23;15(10):e0241140. doi: 10.1371/journal.pone.0241140 (PMC7584171; doi:10.1371/journal.pone.0241140)

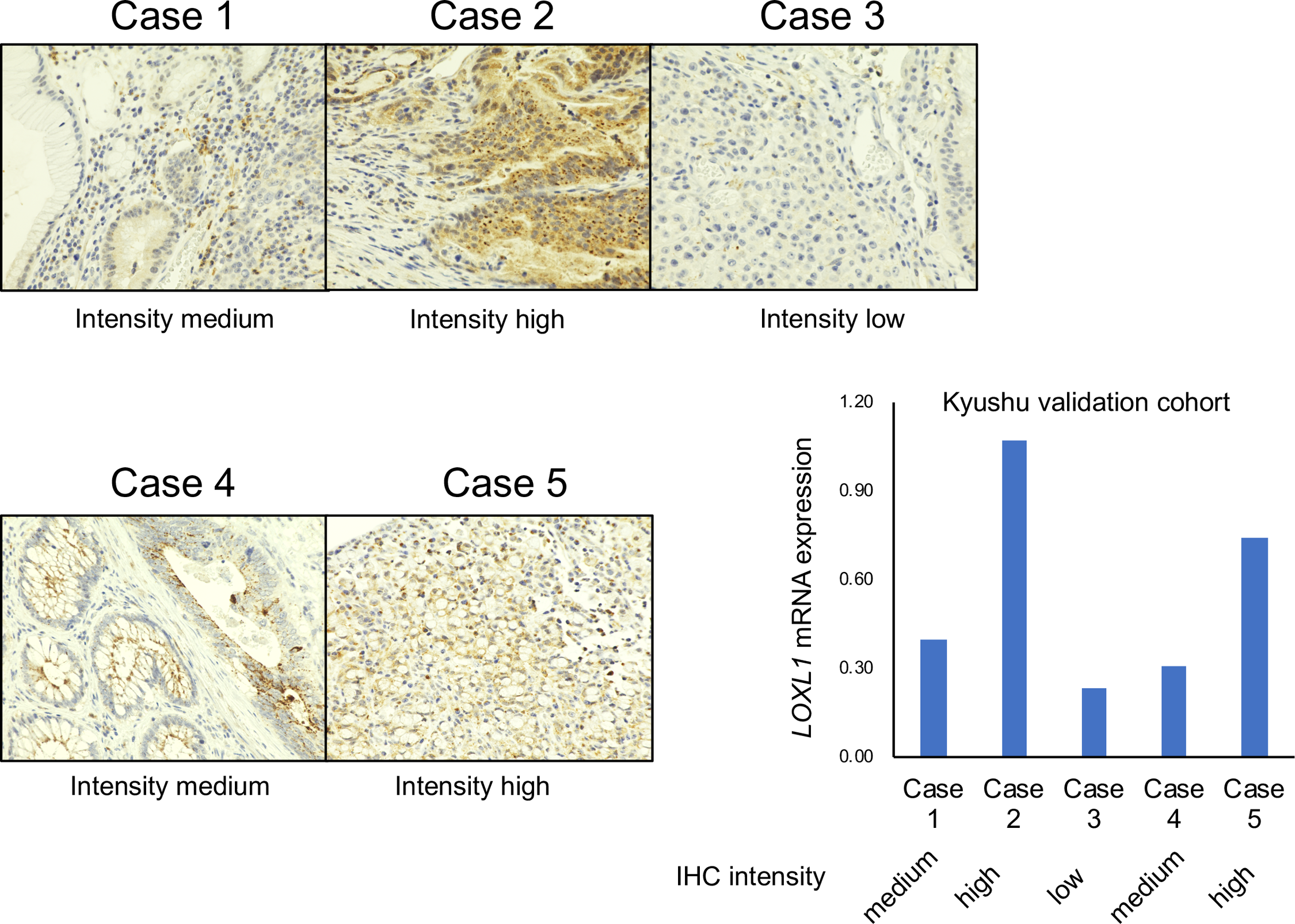

Supplement: S1 Fig — Immunochemistry staining of LOXL1 in 5 GC tissues from the Kyushu validation cohort. Original magnification, ×40, ×100, ×400; LOXL1 immunostaining intensity in tumor cells from the 5 GC tissues were classified into three levels (low, medium, and high). (ZIP) [file pone.0241140.s001.zip › S1_Fig.tif]

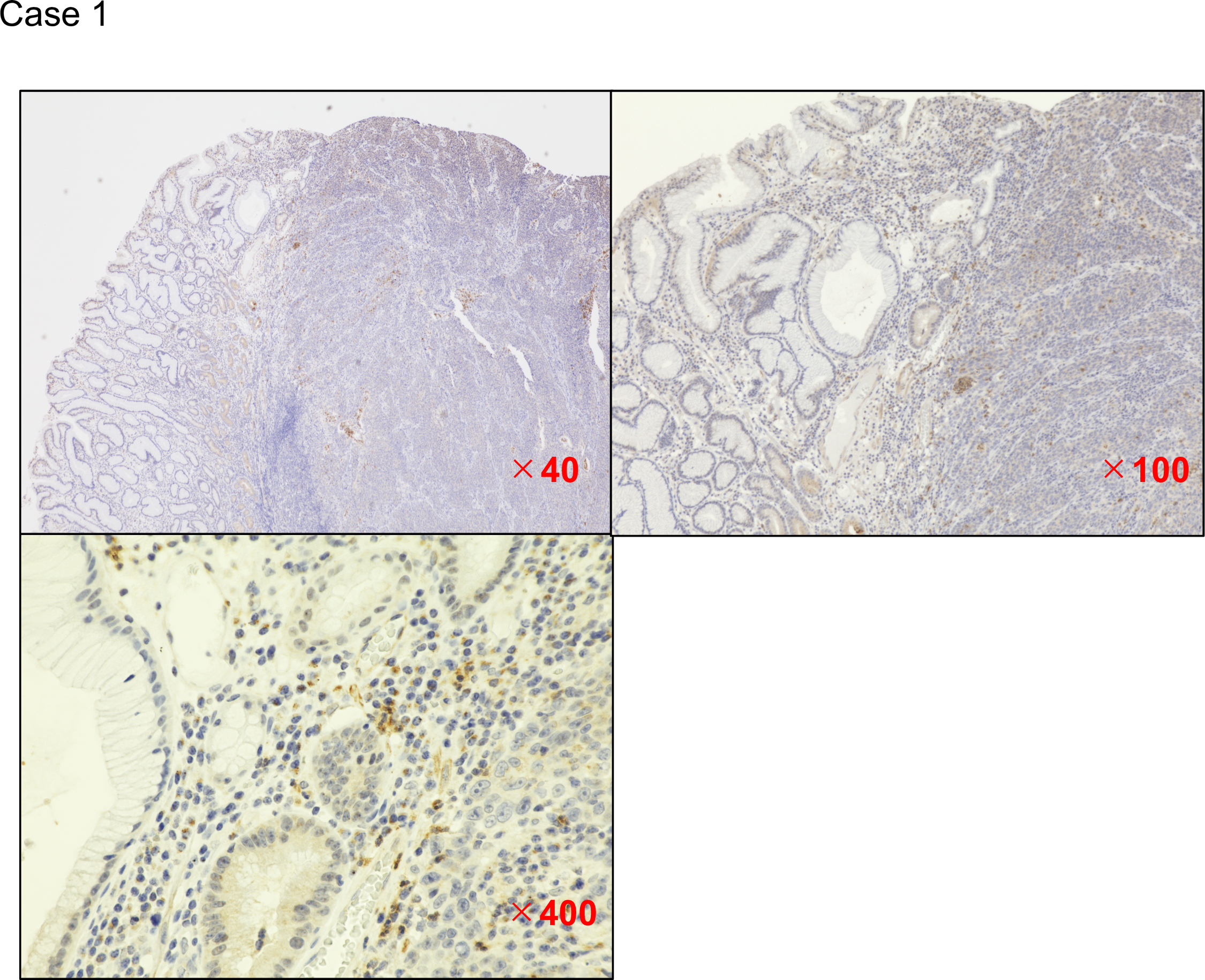

Supplement: S1 Fig — Immunochemistry staining of LOXL1 in 5 GC tissues from the Kyushu validation cohort. Original magnification, ×40, ×100, ×400; LOXL1 immunostaining intensity in tumor cells from the 5 GC tissues were classified into three levels (low, medium, and high). (ZIP) [file pone.0241140.s001.zip › S1_Fig_Case1.tif]

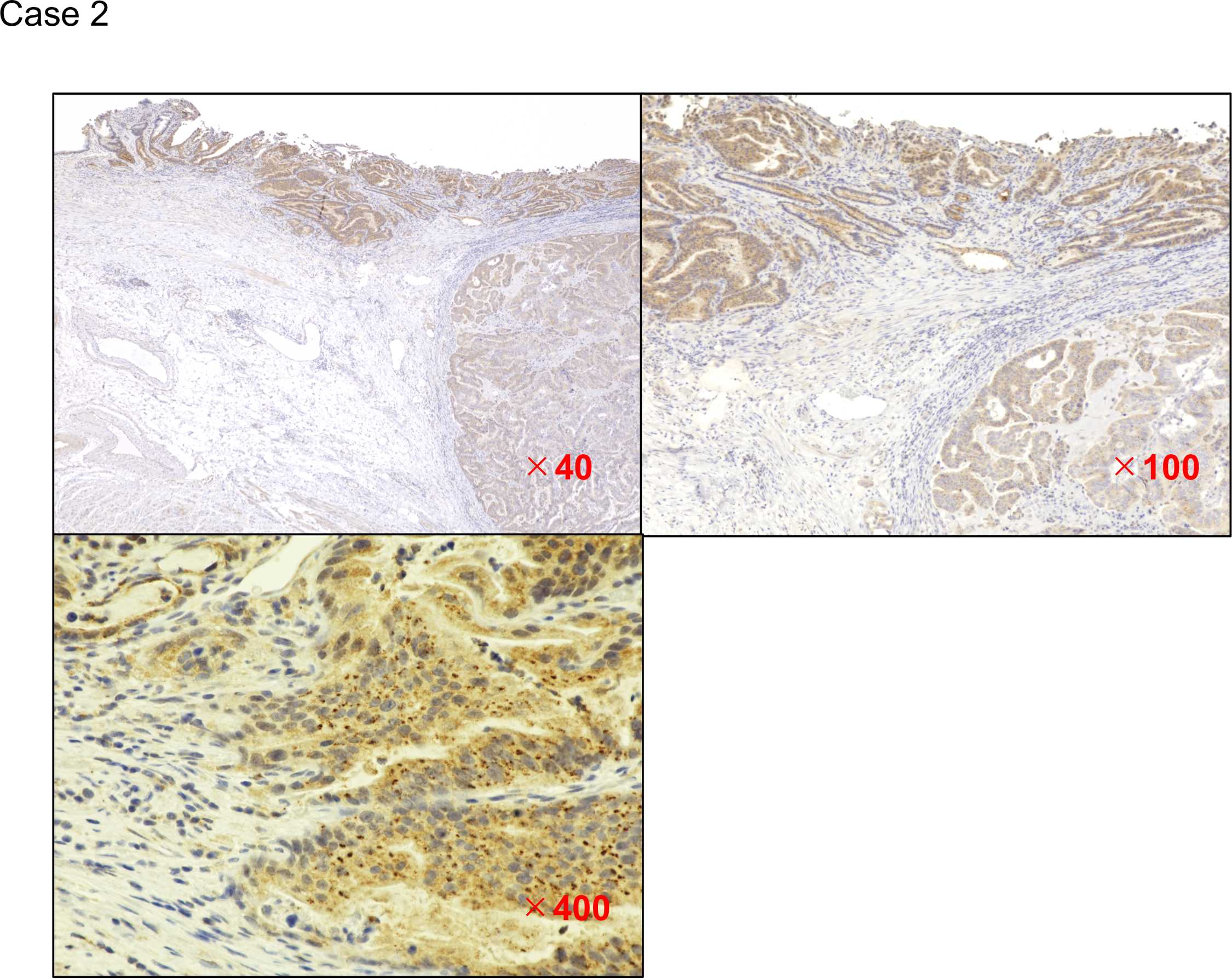

Supplement: S1 Fig — Immunochemistry staining of LOXL1 in 5 GC tissues from the Kyushu validation cohort. Original magnification, ×40, ×100, ×400; LOXL1 immunostaining intensity in tumor cells from the 5 GC tissues were classified into three levels (low, medium, and high). (ZIP) [file pone.0241140.s001.zip › S1_Fig_Case2.tif]

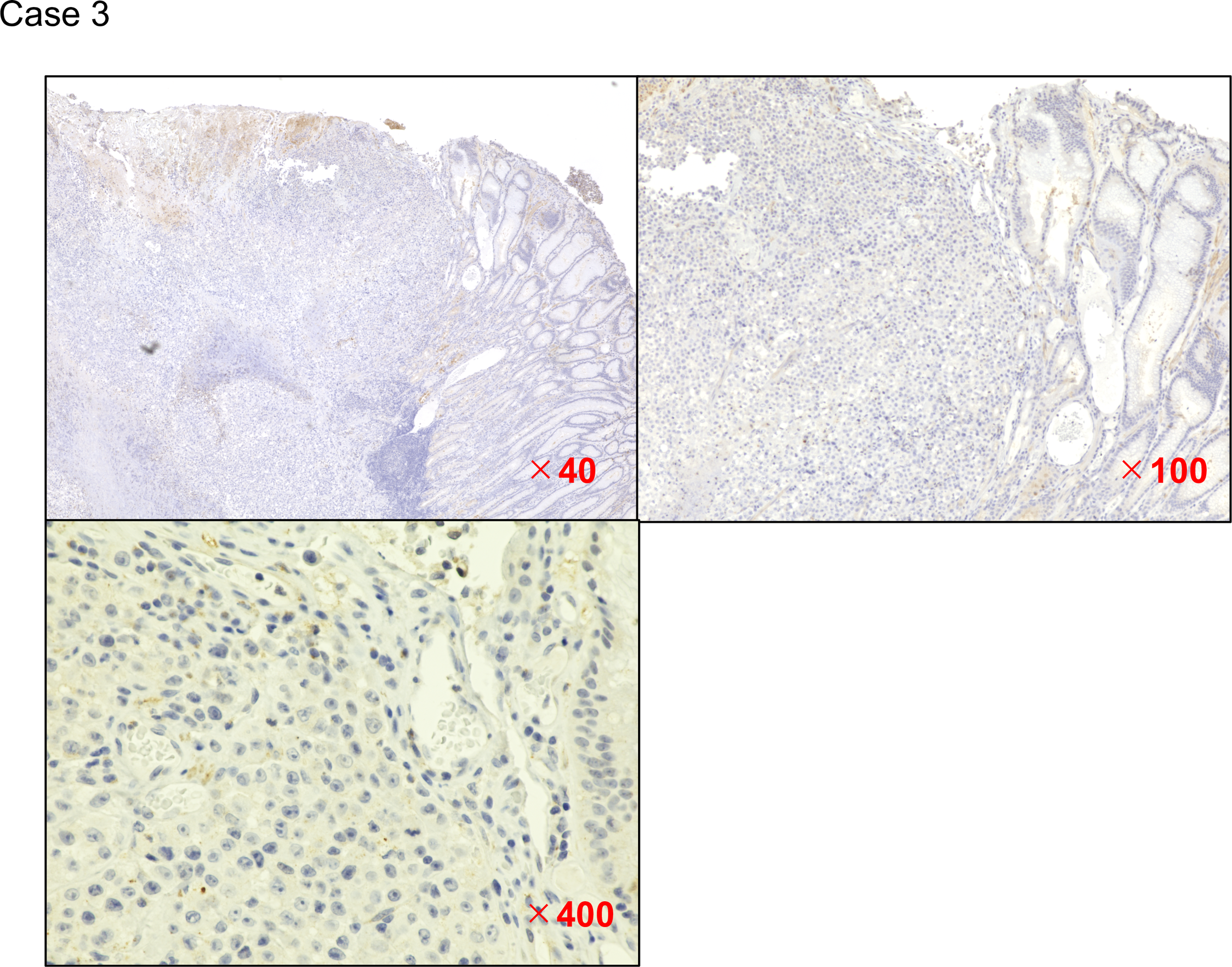

Supplement: S1 Fig — Immunochemistry staining of LOXL1 in 5 GC tissues from the Kyushu validation cohort. Original magnification, ×40, ×100, ×400; LOXL1 immunostaining intensity in tumor cells from the 5 GC tissues were classified into three levels (low, medium, and high). (ZIP) [file pone.0241140.s001.zip › S1_Fig_Case3.tif]

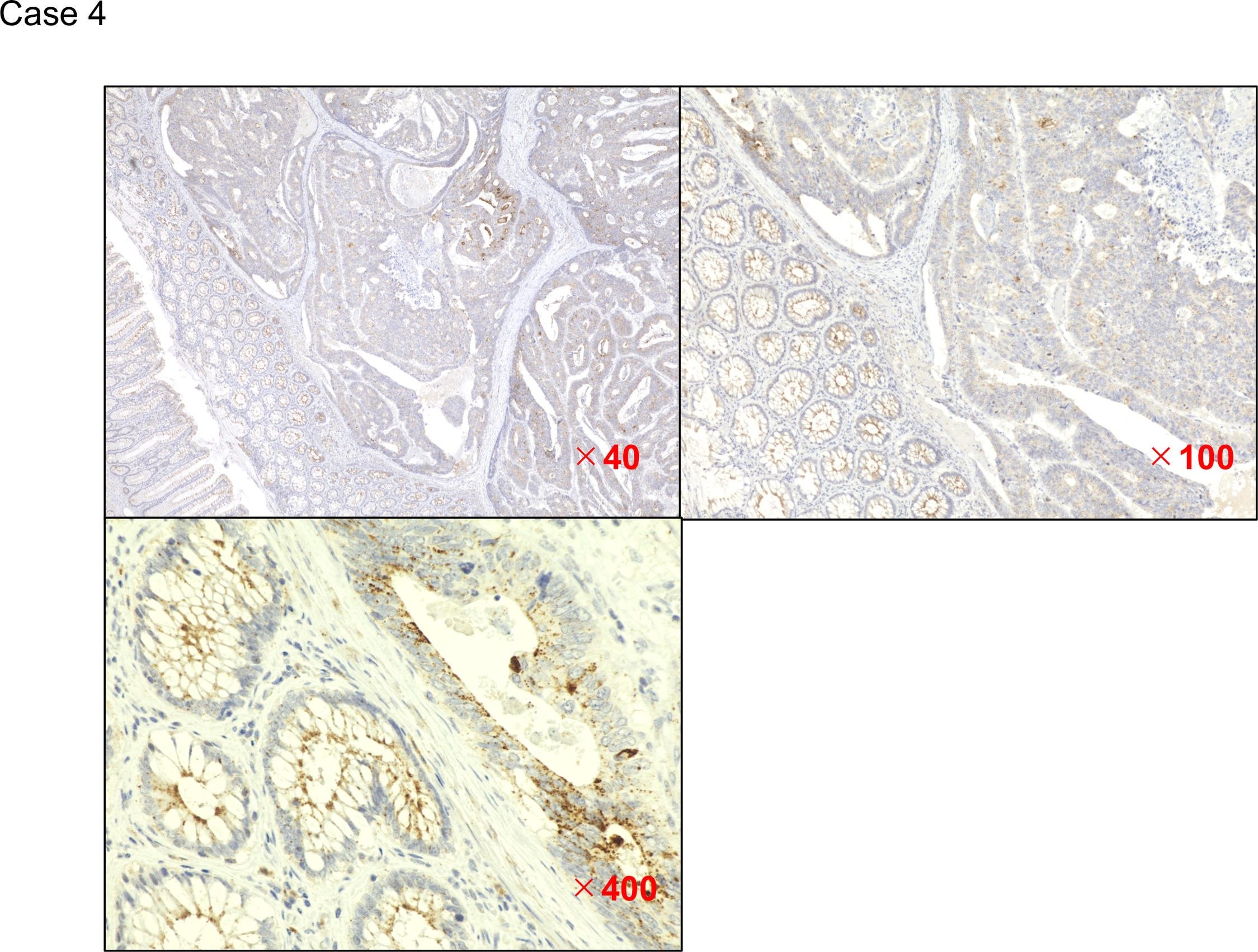

Supplement: S1 Fig — Immunochemistry staining of LOXL1 in 5 GC tissues from the Kyushu validation cohort. Original magnification, ×40, ×100, ×400; LOXL1 immunostaining intensity in tumor cells from the 5 GC tissues were classified into three levels (low, medium, and high). (ZIP) [file pone.0241140.s001.zip › S1_Fig_Case4.tif]

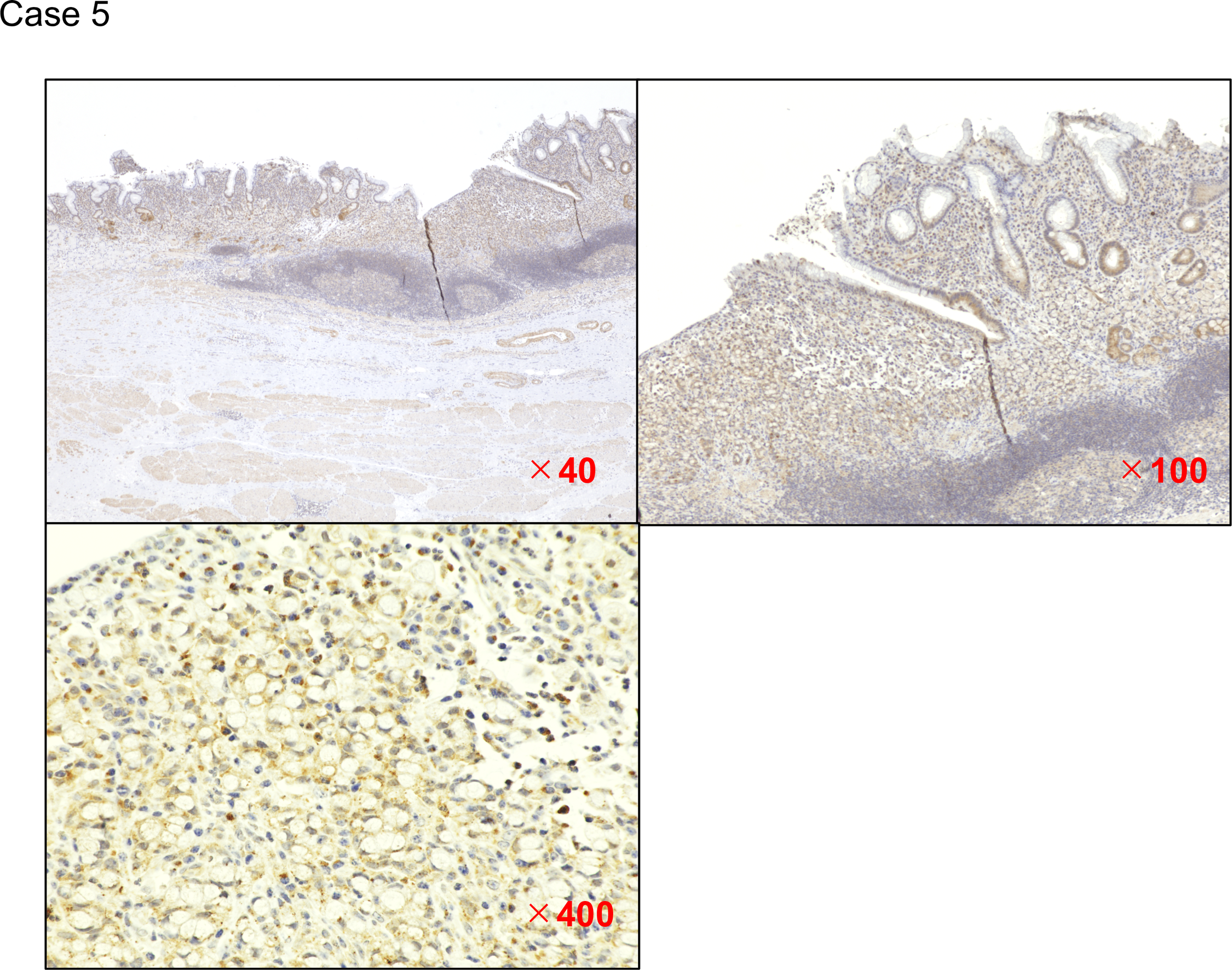

Supplement: S1 Fig — Immunochemistry staining of LOXL1 in 5 GC tissues from the Kyushu validation cohort. Original magnification, ×40, ×100, ×400; LOXL1 immunostaining intensity in tumor cells from the 5 GC tissues were classified into three levels (low, medium, and high). (ZIP) [file pone.0241140.s001.zip › S1_Fig_Case5.tif]

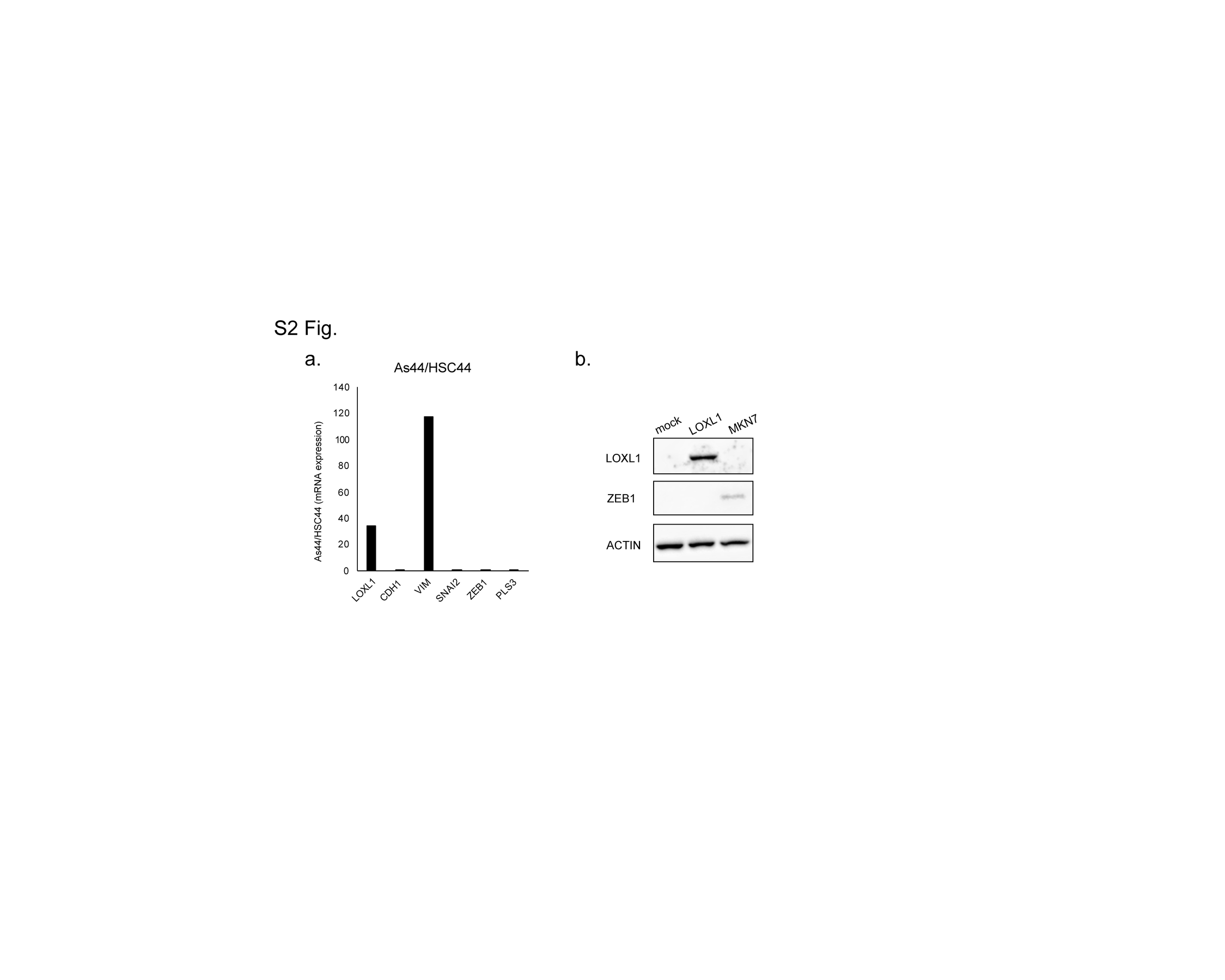

Supplement: S2 Fig — a. The ratios of LOXL1 expression, CDH1 expression, VIM expression, SNAI2 expression, ZEB1 expression, and PLS3 expression in As44 cells relative to HSC44 cells. b. Western blot analysis of LOXL1, ZEB1, and ACTIN protein expression in LOXL1-overexpressing AGS cells, the control AGS cells, and MKN7 cells. (TIF) [file pone.0241140.s002.tif]

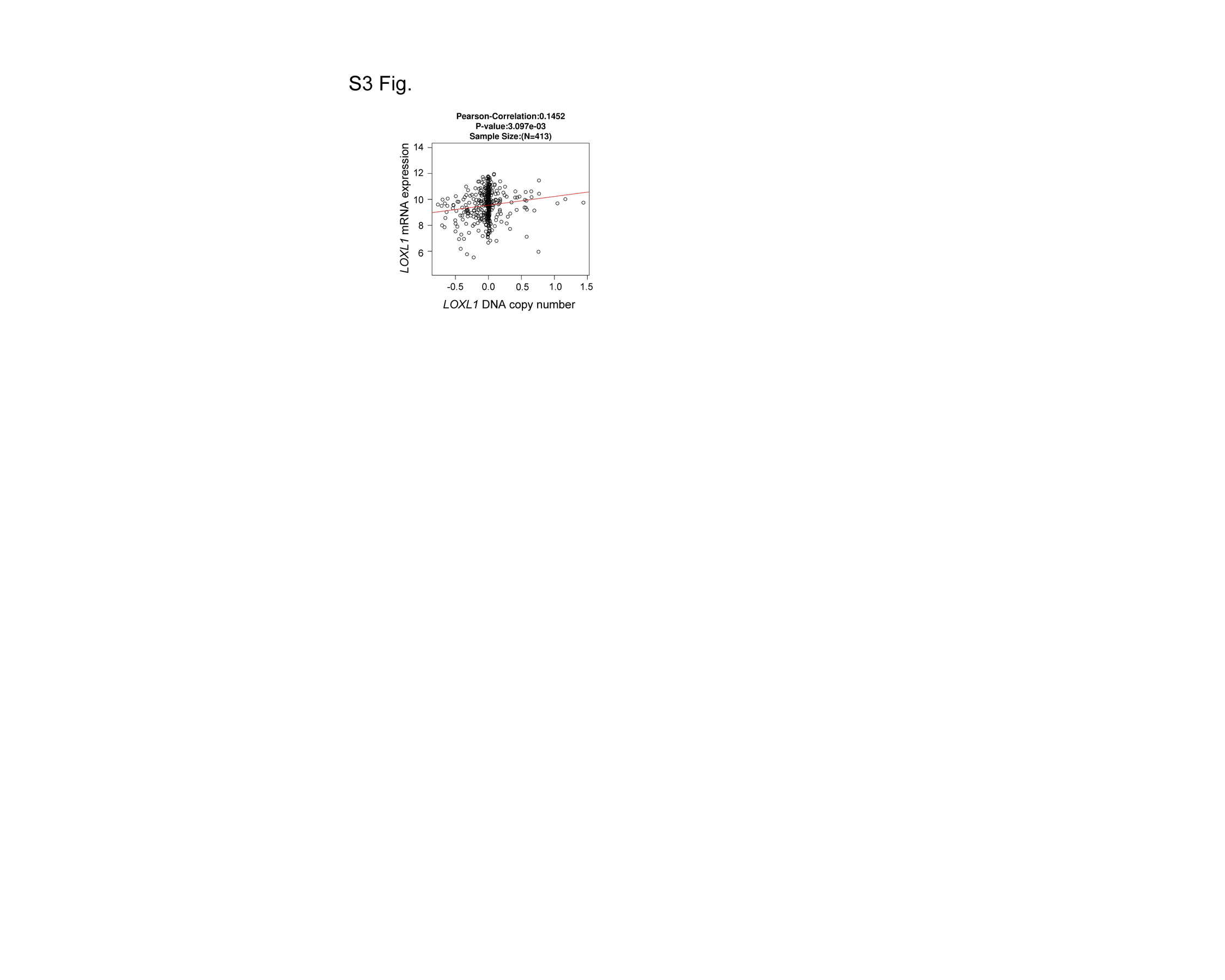

Supplement: S3 Fig — Correlation between LOXL1 mRNA expression and DNA copy number in GC patients. (TIF) [file pone.0241140.s003.tif]

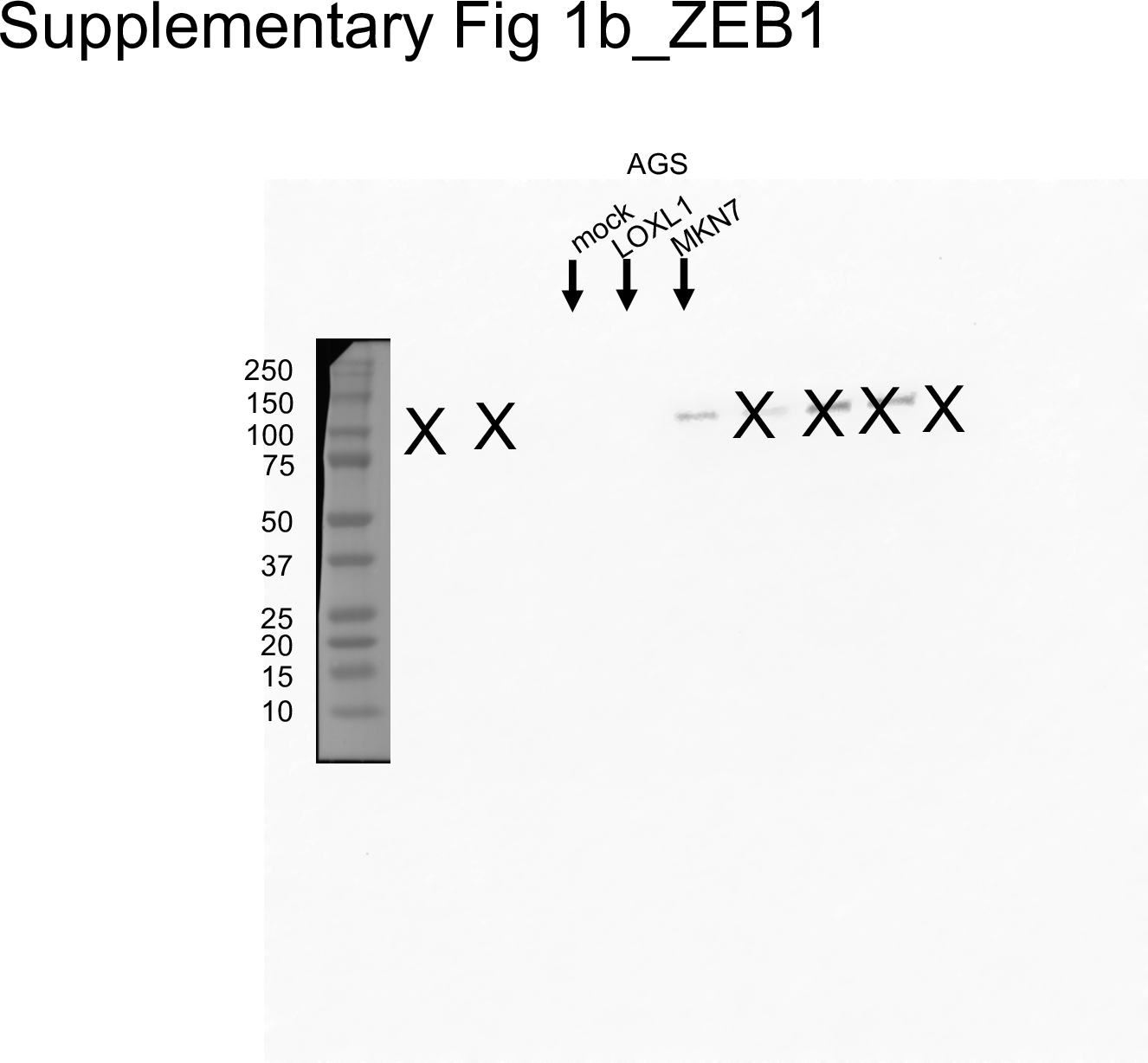

Supplement: S4 Fig — (ZIP) [file pone.0241140.s004.zip › S4_Fig_1.tif]

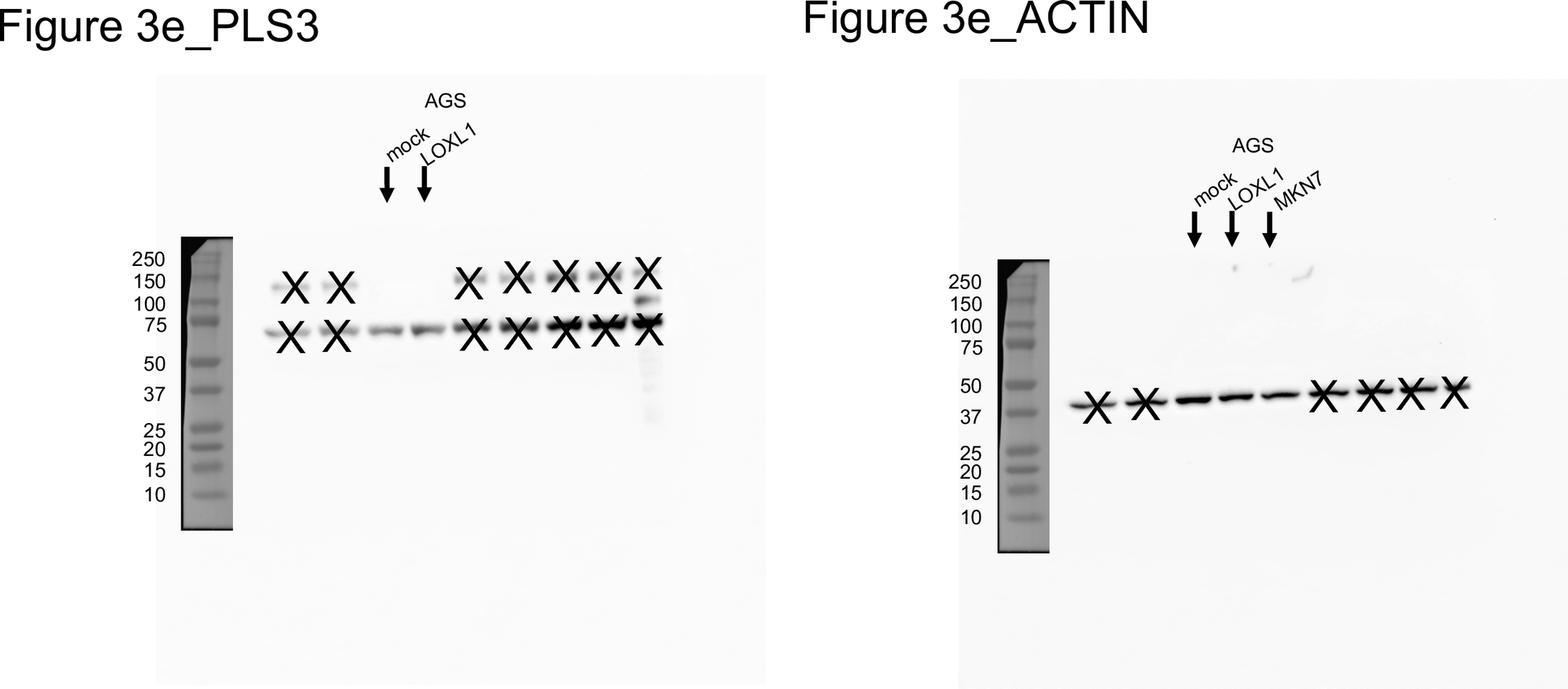

Supplement: S4 Fig — (ZIP) [file pone.0241140.s004.zip › S4_Fig_2.tif]

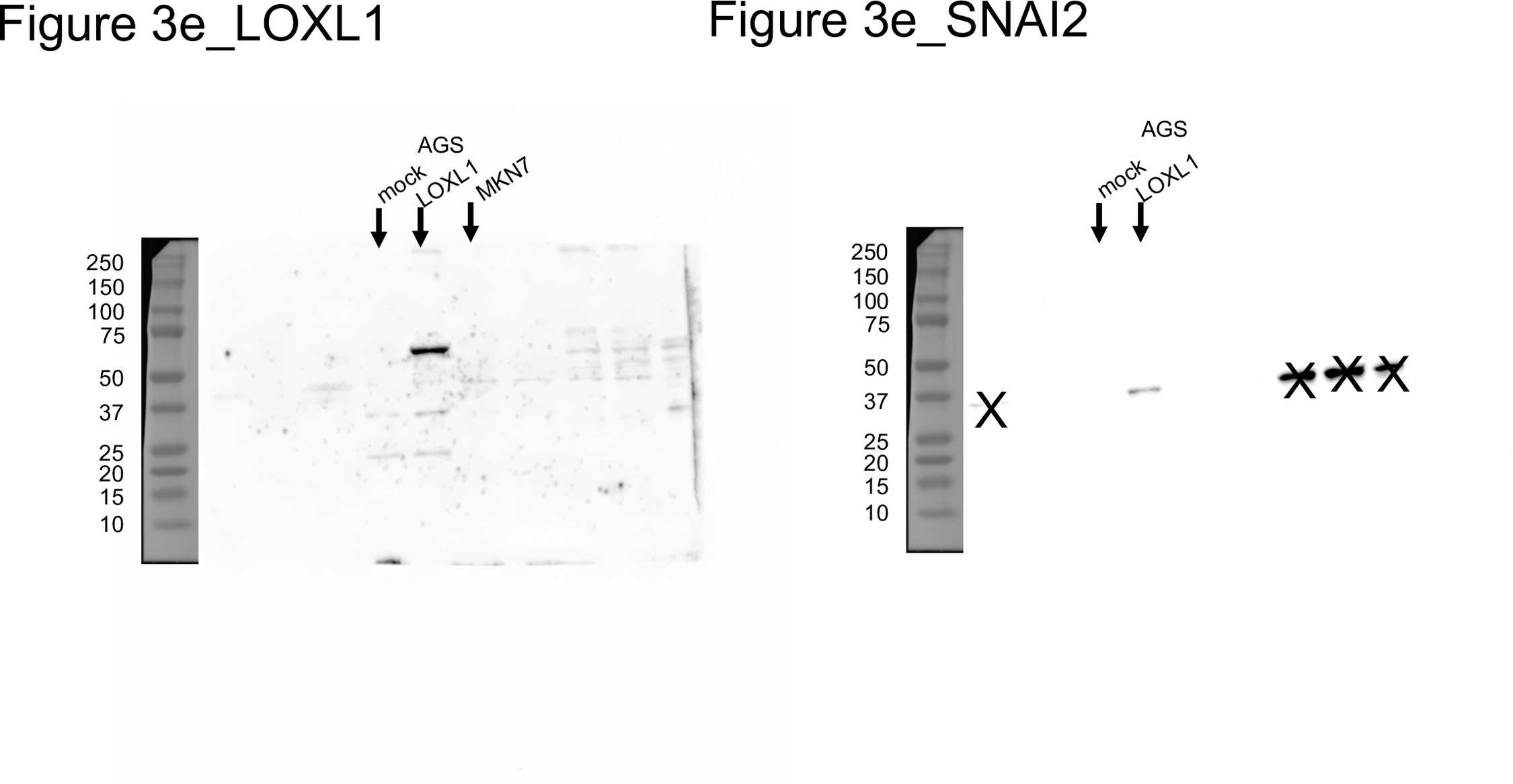

Supplement: S4 Fig — (ZIP) [file pone.0241140.s004.zip › S4_Fig_3.tif]

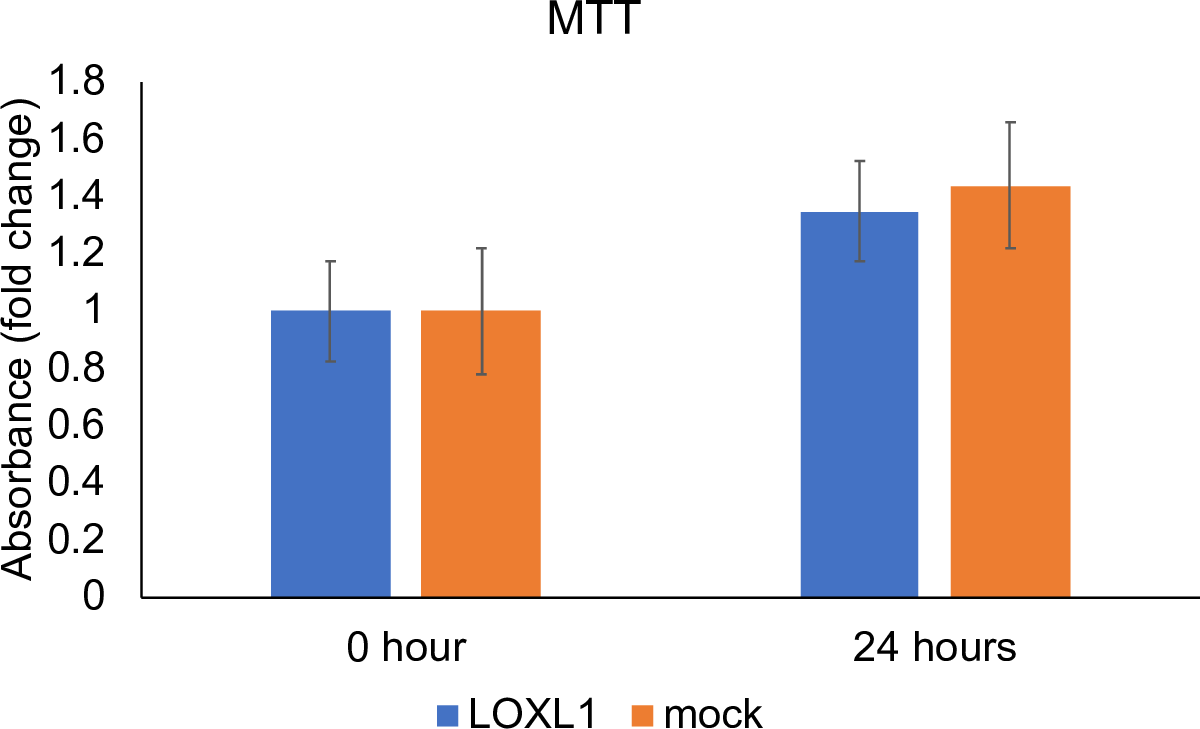

Supplement: S1 File — (TIF) [file pone.0241140.s006.tif]
