# Supplementary material for: Potential association of LOXL1 with peritoneal dissemination in gastric cancer possibly via promotion of EMT
Source: PLoS One. 2020 Oct 23;15(10):e0241140. doi: 10.1371/journal.pone.0241140 (PMC7584171; doi:10.1371/journal.pone.0241140)
Supplement: S1 Table — (DOCX) [file pone.0241140.s005.docx]

**S1 Table. Clinicopathological factors of GC cases with top 10 high and top 10 low *LOXL1* mRNA expression in the Kyushu validation cohort (n = 20, Chi-squared test for P-value).**

| Factors | High expression | Low expression |  |
| --- | --- | --- | --- |
|  | (n = 10) | (n = 10) |  |
|  | Number (%) | Number (%) | P-value |
| Histological type |  |  |  |
| MUC, POR, SIG | 5 (50) | 4 (40) | 0.65 |
| Depth of tumor invasion |  |  |  |
| ≥ SE | 3 (30) | 1 (10) | 0.25 |
| Lymph node metastasis |  |  |  |
| (+) | 8 (80) | 4 (40) | 0.06 |
| Liver metastasis |  |  |  |
| (+) | 1 (10) | 0 (0) | 0.23 |
| Peritoneal dissemination |  |  |  |
| (+) | 1 (10) | 0 (0) | 0.23 |
| Distant metastases |  |  |  |
| (+) | 2 (20) | 1 (10) | 0.53 |
| pStage |  |  |  |
| ≥ III | 7 (70) | 4 (40) | 0.17 |

*SD* standard deviation, *MUC* mucinous adenocarcinoma, *POR* poorly differentiated adenocarcinoma, *SIG* signet-ring cell carcinoma, *SE* serosa
